# Revisiting the Relationship Between Ethnicity and Outcome in Glioblastoma Patients

**DOI:** 10.7759/cureus.954

**Published:** 2017-01-04

**Authors:** Ashish H Shah, Eric Barbarite, Christopher Scoma, Manish Kuchakulla, Sahil Parikh, Amade Bregy, Ricardo J Komotar

**Affiliations:** 1 Department of Neurological Surgery, University of Miami Miller School of Medicine

**Keywords:** ethnicity, prognosis, glioblastoma, outcome

## Abstract

Background: Relationships between various ethnicities and glioma subtype have recently been established. As a tertiary referral center for Latin America and the Caribbean, our institution treats a diverse glioblastoma (GBM) population. We sought to clarify the role of ethnicity on patient prognosis in GBM and also compared these findings to a group consisting of elderly patients. We included ‘elderly’ as a group because the subgroups for ethnicities within them were too small. It allowed us to put in scope the effects of ethnicities on the overall survival.

Material and Methods: After Institutional Review Board approval, 235 patients with GBM were retrospectively identified. A total of 140 patients were separated into four groups: White adults (n = 47), Hispanic adults (n = 27), elderly (n = 58), and Black adults (n = 6). Overall survival (OS) was our primary endpoint.

Results: Overall survival in the White adult group was 24.3 months, compared to 13.0 months in the Hispanic adult group, 20.2 months in the Black group, and 13.8 months in the elderly group (p = 0.01). In the Hispanic group, hypertension (37.9%, p = 0.01) and diabetes (24.1%, p = 0.009) were significantly more prevalent compared to the White adult cohort. No difference in insurance status or postoperative complications was found between subgroups.

Conclusion: Based on our analysis, Hispanic adults may have a decreased survival compared to White adults. However, the incidence of hypertension and diabetes was markedly higher in our Hispanic adult cohort; thus, estimating the risk of ethnicity and comorbidities on patient prognosis may be difficult. A prospective study correlating the genome and subgroup prognosis may help elucidate the role of ethnicity in GBM patients.

## Introduction

Over the last decade, therapy for glioblastoma multiforme (GBM) has been centered on maximal safe surgical resection with combination radiotherapy and adjuvant temozolomide chemotherapy [1]. Despite optimal treatment, the overall five-year survival still remains poor with an average survival of 14 months after diagnosis [1-3]. Specific subsets of patients with unfavorable prognoses, including the elderly, the uninsured, and patients with low preoperative Karnofsky performance scores, have been identified [4-18]. Previously, it had been proposed that ethnicity did not influence prognosis in patients with GBM [3]. However, with the development of microRNA and mutational analyses, relationships between ethnicity and glioma subtype incidence have been identified so far in African-Americans, Whites, and East Asians [19-21]. Nevertheless, a correlation between ethnicity and prognosis in patients with GBM has yet to be identified [22].

As a tertiary referral center for South Florida, the Caribbean, and Latin America, our institution treats a diverse patient population, including numerous Hispanics. As a result, we sought to clarify the relationship between ethnicity and outcome in GBM patients at our institution.

## Materials and methods

The University of Miami Miller School of Medicine Institutional Review Board (IRB# 20110821) approved this study prior to retrospective review of patient data. From 1995-2006, 235 patients with intracranial glioblastoma were identified from an electronic billing database at a single tertiary care referral center. In all cases, a neuropathologist confirmed the diagnosis of glioblastoma using standard diagnostic classification systems (World Health Organization (WHO) Classification System) [23]. All patients were initially screened by age (> 18 years), location (intracranial), and pathology (WHO Grade IV glioma). Patients with multicentric/multifocal GBM were included in the study. For all patients included, an attempt to safely maximize the extent of resection was made when possible. Patients without adequate follow-up/outcome data, operative reports, or hospitalization information were excluded. If follow-up was inadequate (< 6 weeks), patients were excluded.

Patients who did not identify ethnicity/race on clinical documentation were excluded. No attempt was made to assume ethnic or racial data based on the last name.

Relevant variables were obtained, which included sex, age at diagnosis, tumor size (when available), tumor location, the extent of resection, use of adjuvant therapies including chemotherapy/radiotherapy, and follow-up. The extent of resection was determined by either postoperative imaging or by the surgeon’s description in the operative report. Comorbid conditions, including heart disease, diabetes, smoking, hyperlipidemia, and hypertension, were also recorded for each patient when available. Time to recurrence was unable to be assessed in many patients due to lack of imaging data prior to electronic medical records. For patients with adequate follow-up in the clinic, progression-free survival (PFS) was analyzed. Death date was determined primarily by the Social Security database (search.ancestry.com/search/db.aspx?dbid=3693). For select patients, in-hospital death information was obtained directly from electronic medical records. Survival was defined as the time from initial diagnosis to death. Overall survival (OS) was defined as the point in which 50% of patients remained alive after initial diagnosis.

Patients were analyzed separately into three groups: White, Hispanic, and Black (as designated by patient). Once separated, additional subgroups were identified: White adults, Hispanic adults, Black adults, and elderly. We included ‘elderly’ as a group because the subgroups for ethnicities within them were too small. It allowed us to put in scope the effects of ethnicities on the overall survival. All relevant clinical data for each subgroup were collected and analyzed for trends.

### Statistical analysis

Univariate t-tests were used to characterize ethnic subgroups to assess for significant discrepancies in age, incidence of comorbidities, the extent of resection, and use of adjuvant therapy in sizeable sample groups (n > 10). In addition, t-test analysis was performed to analyze the difference in OS and PFS in the various subgroups. P-value < 0.05 was considered statistically significant. SOFA statistics software was used for all statistical analysis (version 1.3.5, AGPL3 license, Paton-Simpson & Associates Ltd, New Zealand). 

## Results

**Figure 1:**
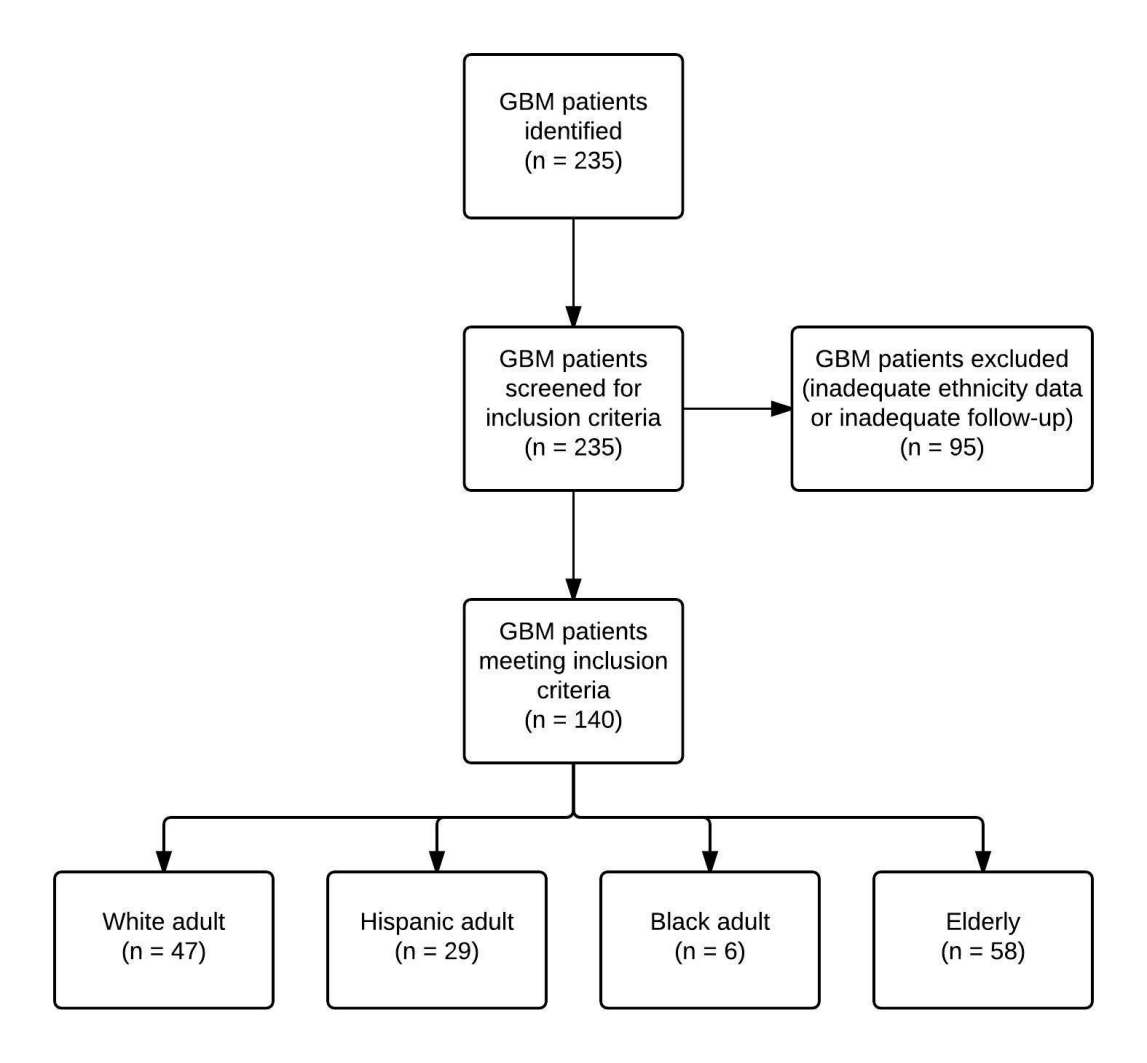
Patient Selection Flow Chart

A total of 140 patients were retrospectively reviewed in our study. Ninety-five patients were excluded due to WHO Grade I-III (n = 79) or inadequate follow-up (n = 16). Included patients were split into four groups (White adults, Hispanic adults, Black adults, and elderly). The identification of patients with GBM and exclusion criteria is described in Figure 1. The White adult category was designated as the control group in our study to compare prognostic variables. In the White adult category (n = 47), the mean age was 50 years. Overall, there was no statistical difference in mean age between the different patient groups (p > 0.05). In the control group, the tumor locations (frontal, parietal, occipital, temporal, etc.) were recorded, which are reported in Table 1, and no statistical difference in tumor location was found between the ethnic groups (p > 0.05). Presenting symptoms within the control group ranged from altered mental status (42.5%), headache (55.3%), motor deficit (23.4%), seizure (29.7%), visual impairment (12.75%), or uncontrolled nausea/vomiting (10.6%). A summary of presenting symptoms between ethnic groups is summarized in Table 1. Tumor size was not recorded in this study.

**Table 1:** Patient Demographics P-value performed as a measure of statistical variance between White adults and Hispanic adults only. Chi-squared test was performed to obtain p-values.

	White Adults (n = 47)	Hispanic Adults (n = 29)	Black Adults (n = 6)	Elderly (n = 58)	P-value*
Average Age, Yrs (n)	50.02	53.586	48.33	74.86	.161
GBM Location					.169
Frontal	22.81%	34.29%	14.29%	35.82%	--
Parietal	17.54%	14.29%	42.86%	19.4%	--
Occipital	8.77%	8.57%	14.29%	10.45%	--
Temporal	35.09%	34.29%	34.29%	29.85%	--
Thalamic	1.75%	2.86%	0%	0%	--
Other	14.04%	5.71%	0%	4.48%	--
Presenting Symptom				
Altered mental status	42.5%	51.7%	50%	55.1%	0.0573
Headache	55.3%	48.2%	66.6%	25.8%	0.573
Hemiparesis	23.4%	41.3%	33.3%	37.9%	0.203
Seizure	29.7%	20.6%	50%	18.9%	0.474
Visual impairment	12.7%	20.6%	0%	13.7%	0.496
Vomiting/Nausea	10.6%	24.1%	50%	3.4%	0.227

### Comorbidities

Various common comorbidities were assessed in each ethnic group, including coronary artery disease, hyperlipidemia, smoking history, hypertension, diabetes mellitus (both insulin-dependent and non-insulin dependent), and human immunodeficiency virus (HIV) infection. In the control group, smoking and hypertension were the most common comorbid conditions (21.2% and 12.7%, respectively). In the Hispanic adult cohort, hypertension (37.9%, p = 0.01) and diabetes (24.1%, p = 0.009) were significantly more prevalent compared to the White adult cohort. A summary of relevant comorbidities for each patient cohort is reported in Table 2. No other statistically significant difference was found for other comorbidities. 

**Table 2:** Patient Comorbidities P-value performed as a measure of statistical variance between White Adults and Hispanic Adults only. Chi-squared test was performed to obtain p-values.

	White Adults (n = 47)	Hispanic Adults (n = 29)	Black Adults (n = 6)	Elderly (n = 58)	P-value*
Comorbidities					
Coronary artery disease	2.1%	0%	0%	13.7%	0.429
Hyperlipidemia	4.2%	6.8%	0%	15.5%	0.616
History of Smoking	21.2%	6.8%	16.6%	24.1%	0.771
Hypertension	12.7%	24.1%	16.6%	48.2%	0.011
Diabetes Mellitus	4.2%	24.1%	33.3%	25.8%	0.009
HIV	2.1%	0%	0%	0%	0.429

### Operative complications

All patients in our study were followed-up at least six weeks after surgery until death.

Postoperative morbidity was reported for each ethnic group in Table 3. Common complications included aphasia, altered mental status, motor deficit, infection, stroke, and gastrointestinal symptoms, such as nausea/vomiting. The most common postoperative complication within all patient groups was motor deficits. No statistical significance was found between patient groups.

**Table 3:** Postoperative Complications

Postop Complication	White Adults (n = 47)	Hispanic Adults (n = 29)	Black Adults (n = 6)	Elderly (n = 58)
Aphasia	0%	3.4%	0%	1.7%
Confusion	2.1%	3.4%	0%	5.2%
Motor deficit	6.3%	6.8%	0%	1.7%
Infection	0%	3.4%	0%	0%
Stroke	2.1%	0%	0%	0%
Gastrointestinal	2.1%	0%	0%	3.5%

### Outcomes

**Figure 2:**
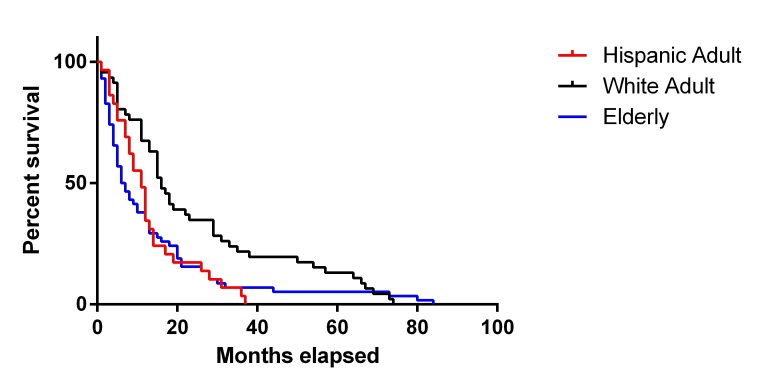
Overall Survival Kaplan-Meier Curve

**Figure 3:**
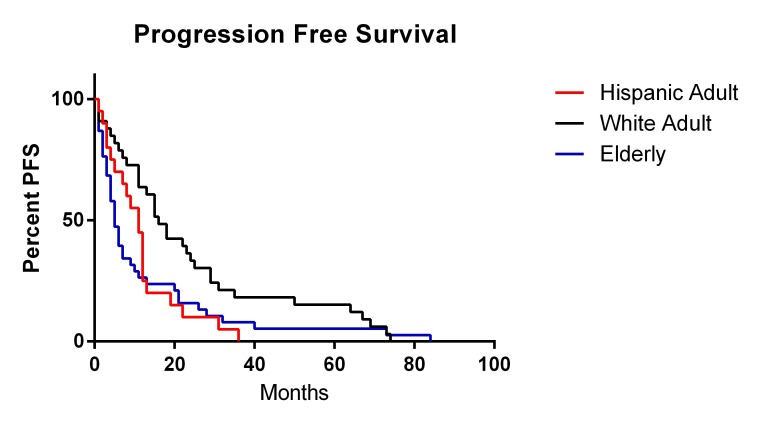
Progression-Free Survival Kaplan-Meier Curve

Overall survival for each ethnic group was reported in Table 4. For the White adult group, overall survival after diagnosis was 24.3 months. Overall survival within our Hispanic adult cohort was significantly decreased compared to the White adult group (OS = 13.0, p = 0.01). The elderly patient cohort had a similar overall survival of 13.8 months. Kaplan-Meier curves for OS and progression-free survival (PFS) are reported in Figures 2-3, respectively. The Hispanic adult group had an overall lower OS and PFS compared to the White adult group. Given the exclusion criteria for follow-up in our retrospective analysis (less than six weeks), only six patients were ultimately included in the Black patient cohort. While the demographic, comorbidity, and postoperative complication data of the Black patient cohort are represented in Tables 1-3, only a fraction of these patients had sufficient data to determine overall survival. As a result, the power of our statistical analysis based on such a small number was low, and overall survival of the Black patient cohort was ultimately not calculated.

**Table 4:** Patient Overall Survival Overall survival is expressed here in months. P-value performed as a measure of statistical variance between White Adults and Hispanic Adults only. Independent t-test was performed for patient groups against OS.

	White Adults (n = 47)	Hispanic Adults (n = 29)	Black Adults (n = 6)	Elderly (n = 58)	P-value
Overall Survival	24.3	13.0	20.2	13.8	0.01

### Insurance status

Patients in our study possessed private insurance, Medicaid, Medicare, or received health care using a local public provider. There was no statistical difference in patients who possessed private insurance or public/low-cost insurance providers (Medicaid, local public provider) between all ethnic cohorts in our study.

## Discussion

Patients with GBM have a median overall survival of approximately 14 months after surgical resection and concomitant radiochemotherapy [1]. Despite this dismal survival rate, certain prognostic factors have been associated with improved OS in select patient populations. Although many clinical studies compare factors determined postoperatively (extent of resection, chemotherapy, radiotherapy, or MGMT (O-6-methylguanine-DNA methyltransferase) status), few studies have isolated prognosticators in patient demographics, such as ethnicity, gender, and the presence of comorbidities. Published data on preoperative negative prognosticators exists for the elderly GBM patients or patients with large butterfly GBMs [9, 12-13, 24-29]. Nevertheless, data suggesting a relationship between race/ethnicity and prognosis in GBM patients is scarce. For example, select studies in homogeneous single-country ethnic groups (China) have failed to demonstrate significant survival differences from reported literature [3]. Yet, other studies suggest that non-Latino whites are at an increased risk of developing GBM [30]. Although a survival disadvantage has not been linked to specific race/ethnicity as of yet, further research in ethnically diverse centers may reveal the prognostic significance of ethnicity in highly malignant GBM. As a tertiary care center located in one of the most diverse areas in the United States, the University of Miami Miller School of Medicine was poised to adequately assess this.

### Survival

In this study of 140 patients with confirmed WHO Grade IV glioblastoma, overall survival was 9.3 months less within our Hispanic adult group (ages: 18 - 65) compared to a cohort of White adults (ages: 18 - 65). The difference in OS between the White adult and Hispanic adult groups was statistically significant (p = 0.01). There were no significant differences in postoperative complications, insurance status, location, or presenting symptoms between the ethnic cohorts. All patients in our study were given similar treatments (surgery + adjuvant radiation + concomitant chemotherapy), and no differences in treatment options were discovered. However, in multivariate analysis, hypertension and diabetes were more prevalent in the Hispanic adult patient group compared to other groups.

### Comorbidities

In our diverse patient population, the incidence of diabetes, coronary artery disease risk factors, and hypertension may be elevated in certain minority populations [31-32]. The reasons for such health discrepancies may be multifactorial: socioeconomic status, access to care, dietary differences, cultural resistance to allopathic medicine, and genetic predisposition [33-36]. Previously, it has been shown that the presence of comorbidities in the elderly GBM population may influence the treatment effect and, ultimately, OS [9, 37]. However, a relationship between OS and presence of comorbidities in the general adult GBM population has not been clear. In our study, the presence of more comorbidities in the Hispanic adult group may confound our primary outcomes; nevertheless, such data may give us insight on yet another preoperative risk factor within select patient populations. Due to the nature of our study and the confounding factors, an explanation for a survival disadvantage within Hispanic adults could not be found.

### Limiting factors

Our study was performed as a retrospective review of select patients treated at our institution over the last 10 years for whom full data sets were available. Although we were able to isolate data from well over 140 patients with histologically confirmed GBM, several patients were not included in our study due to lack of follow-up, an incomplete search strategy, and an absence of identifiable information. The latter may explain the paucity of Black adults (n = 6) in our study. After accounting for these limitations, our patient population was nevertheless adequate for statistical analysis. 

Secondly, because our study was conducted as a retrospective review, the true attributable death risk of Hispanic adults with GBM could not be calculated. Additionally, the presence of insurance status remains another potential confounding factor that may influence outcomes in patients with GBM. Previously, it has been demonstrated that healthcare status affects outcomes in patients with brain tumors [38-39]. However, in our study, no clear difference in insurance status was found, which may, largely in part, be due to the presence of a public healthcare provider (Jackson Memorial Hospital), the largest public county hospital in the United States. Within this system, patients without insurance can obtain surgery, radiation, and chemotherapy for GBM. Furthermore, in our study, we were unable to obtain income or other socioeconomic data points from our study group. Therefore, a thorough analysis of other socioeconomic risk factors may be obscured.

Lastly, we recognize an important factor in any discussion on diverse ethnic groups, such as Hispanics, since the Hispanic subset of patients in Miami can be largely variable across every socioeconomic group and nationality. Therefore, comparing socioeconomic classes in a subset of specific Hispanics may be important in teasing out the true prognostic differences among our patient group. In our county hospital, the majority of patients arrive from lower socioeconomic groups; yet, because we remain a tertiary referral center, a significant portion of our patients is private as well. Further epidemiological research in a broader study may be helpful to understand the role of socioeconomic class or ethnicity on GBM prognosis.

### Future studies

In order to effectively assess healthcare disparities among the Hispanic adult GBM population, we have begun prospectively enrolling patients into a clinical database that will allow for outcome analyses of select ethnic groups. Using this IRB-approved database, complete patient information, including insurance status, self-reported ethnicity, and postoperative complications, can be directly obtained without the inherent biases of retrospective studies. Additionally, by collecting histological specimens simultaneously, genomic profiles of various ethnic subpopulations could be correlated to patient prognosis.

## Conclusions

Based on our retrospective analysis of 140 GBM patients, Hispanic adults (age: 18 - 65 years) may have a decreased survival compared to age-matched controls. However, in this Hispanic adult cohort, the incidence of hypertension and diabetes was markedly higher than other groups; therefore, estimating the risk of ethnicity/comorbidities on patient prognosis might be difficult. This remains one of the first studies in adults to show survival differences in certain ethnic subpopulations for GBM. At present, conclusive evidence on the survival disadvantage of Hispanic adults is not present, and further prospective studies are needed to fully assess the socioeconomic risk factors, comorbidities, and genetic predisposition in certain ethnic subgroups for GBM. Although our study does not definitively verify ethnicity as a negative prognosticator, it suggests the heterogeneous nature of GBM and the need for subpopulation-specific research studies.

## References

[1] Stupp R, Hegi ME, Mason WP, van den Bent MJ, Taphoorn MJ, Janzer RC, Ludwin SK, Allgeier A, Fisher B, Belanger K, Hau P, Brandes AA, Gijtenbeek J, Marosi C, Vecht CJ, Mokhtari K, Wesseling P, Villa S, Eisenhauer E, Gorlia T, Weller M, Lacombe D, Cairncross JG, Mirimanoff RO; European Organisation for Research and Treatment of Cancer Brain Tumour and Radiation Oncology Groups; National Cancer Institute of Canada Clinical Trials Group (2009). Effects of radiotherapy with concomitant and adjuvant temozolomide versus radiotherapy alone on survival in glioblastoma in a randomised phase III study: 5-year analysis of the EORTC-NCIC trial. Lancet Oncol.

[2] Coffey RJ, Lunsford LD, Taylor FH (1988). Survival after stereotactic biopsy of malignant gliomas. Neurosurgery.

[3] Ma X, Lv Y, Liu J, Wang D, Huang Q, Wang X, Li G, Xu S, Li X (2009). Survival analysis of 205 patients with glioblastoma multiforme: clinical characteristics, treatment and prognosis in China. J Clin Neurosci.

[4] Allahdini F, Amirjamshidi A, Reza-Zarei M, Abdollahi M (2010). Evaluating the prognostic factors effective on the outcome of patients with glioblastoma multiformis: does maximal resection of the tumor lengthen the median survival?. World Neurosurg.

[5] Barker FG 2nd, Chang SM, Larson DA, Sneed PK, Wara WM, Wilson CB, Prados MD (2001). Age and radiation response in glioblastoma multiforme. Neurosurgery.

[6] Barnholtz-Sloan JS, Williams VL, Maldonado JL, Shahani D, Stockwell HG, Chamberlain M, Sloan AE (2008). Patterns of care and outcomes among elderly individuals with primary malignant astrocytoma. J Neurosurg.

[7] Borg N, Guilfoyle MR, Greenberg DC, Watts C, Thomson S (2011). Serum albumin and survival in glioblastoma multiforme. J Neurooncol.

[8] Bredel M (2008). Nomograms as clinicobiological predictors of survival in glioblastoma. Lancet Oncol.

[9] Chaichana KL, Chaichana KK, Olivi A, Weingart JD, Bennett R, Brem H, Quiñones-Hinojosa A (2011). Surgical outcomes for older patients with glioblastoma multiforme: preoperative factors associated with decreased survival. Clinical article. J Neurosurg.

[10] Colman H, Aldape K (2008). Molecular predictors in glioblastoma: toward personalized therapy. Arch Neurol.

[11] Colman H, Zhang L, Sulman EP, McDonald JM, Shooshtari NL, Rivera A, Popoff S, Nutt CL, Louis DN, Cairncross JG, Gilbert MR, Phillips HS, Mehta MP, Chakravarti A, Pelloski CE, Bhat K, Feuerstein BG, Jenkins RB, Aldape K (2010). A multigene predictor of outcome in glioblastoma. Neuro Oncol.

[12] Ewelt C, Goeppert M, Rapp M, Steiger HJ, Stummer W, Sabel M (2011). Glioblastoma multiforme of the elderly: the prognostic effect of resection on survival. J Neurooncol.

[13] Fazeny-Dörner B, Wenzel C, Veitl M, Piribauer M, Rössler K, Dieckmann K, Ungersböck K, Marosi C (2003). Survival and prognostic factors of patients with unresectable glioblastoma multiforme. Anticancer Drugs.

[14] Hoshino T, Ahn D, Prados MD, Lamborn K, Wilson CB (1993). Prognostic significance of the proliferative potential of intracranial gliomas measured by bromodeoxyuridine labeling. Int J Cancer.

[15] Keles GE, Lamborn KR, Chang SM, Prados MD, Berger MS (2004). Volume of residual disease as a predictor of outcome in adult patients with recurrent supratentorial glioblastomas multiforme who are undergoing chemotherapy. J Neurosurg.

[16] Miller PJ, Hassanein RS, Giri PG, Kimler BF, O'Boynick P, Evans RG (1990). Univariate and multivariate statistical analysis of high-grade gliomas: the relationship of radiation dose and other prognostic factors. Int J Radiat Oncol Biol Phys.

[17] Senger D, Cairncross JG, Forsyth PA (2003). Long-term survivors of glioblastoma: statistical aberration or important unrecognized molecular subtype?. Cancer J.

[18] Veilleux N, Goffaux P, Boudrias M, Mathieu D, Daigle K, Fortin D (2010). Quality of life in neurooncology--age matters. J Neurosurg.

[19] Jacobs DI, Walsh KM, Wrensch M, Wiencke J, Jenkins R, Houlston RS, Bondy M, Simon M, Sanson M, Gousias K, Schramm J, Labussière M, Di Stefano AL, Wichmann HE, Müller-Nurasyid M, Schreiber S, Franke A, Moebus S, Eisele L, Dewan AT, Dubrow R (2012). Leveraging ethnic group incidence variation to investigate genetic susceptibility to glioma: a novel candidate SNP approach. Front Genet.

[20] Tang J, Shao W, Dorak MT, Li Y, Miike R, Lobashevsky E, Wiencke JK, Wrensch M, Kaslow RA, Cobbs CS (2005). Positive and negative associations of human leukocyte antigen variants with the onset and prognosis of adult glioblastoma multiforme. Cancer Epidemiol Biomarkers Prev.

[21] Yan W, Zhang W, You G, Zhang J, Han L, Bao Z, Wang Y, Liu Y, Jiang C, Kang C, You Y, Jiang T (2012). Molecular classification of gliomas based on whole genome gene expression: a systematic report of 225 samples from the Chinese Glioma Cooperative Group. Neuro Oncol.

[22] Barnholtz-Sloan JS, Maldonado JL, Williams VL, Curry WT, Rodkey EA, Barker FG 2nd, Sloan AE (2007). Racial/ethnic differences in survival among elderly patients with a primary glioblastoma. J Neurooncol.

[23] Kleihues P, Louis DN, Scheithauer BW, Rorke LB, Reifenberger G, Burger PC, Cavenee WK (2002). The WHO classification of tumors of the nervous system. J Neuropathol Exp Neuro.

[24] Hammoud MA, Sawaya R, Shi W, Thall PF, Leeds NE (1996). Prognostic significance of preoperative MRI scans in glioblastoma multiforme. J Neurooncol.

[25] Iwamoto FM, Cooper AR, Reiner AS, Nayak L, Abrey LE (2009). Glioblastoma in the elderly: the Memorial Sloan-Kettering Cancer Center Experience (1997-2007). Cancer.

[26] Iwamoto FM, Reiner AS, Nayak L, Panageas KS, Elkin EB, Abrey LE (2009). Prognosis and patterns of care in elderly patients with glioma. Cancer.

[27] Mohan DS, Suh JH, Phan JL, Kupelian PA, Cohen BH, Barnett GH (1998). Outcome in elderly patients undergoing definitive surgery and radiation therapy for supratentorial glioblastoma multiforme at a tertiary care institution. Int J Radiat Oncol Biol Phys.

[28] Showalter TN, Andrel J, Andrews DW, Curran WJ Jr, Daskalakis C, Werner-Wasik M (2007). Multifocal glioblastoma multiforme: prognostic factors and patterns of progression. Int J Radiat Oncol Biol Phys.

[29] Chaudhry NS, Shah AH, Ferraro N, Snelling BM, Bregy A, Madhavan K, Komotar RJ (2013). Predictors of long-term survival in patients with glioblastoma multiforme: advancements from the last quarter century. Cancer Invest.

[30] Chakrabarti I, Cockburn M, Cozen W, Wang YP, Preston-Martin S (2005). A population-based description of glioblastoma multiforme in Los Angeles County, 1974-1999. Cancer.

[31] Romano JG, Arauz A, Koch S, Dong C, Marquez JM, Artigas C, Merlos M, Hernandez B, Roa LF, Rundek T, Sacco RL (2013). Disparities in stroke type and vascular risk factors between 2 Hispanic populations in Miami and Mexico city. J Stroke Cerebrovasc Dis.

[32] Zarini GG, Exebio JC, Gundupalli D, Nath S, Huffman FG (2011). Hypertension, poor glycemic control, and microalbuminuria in Cuban Americans with type 2 diabetes. Int J Nephrol Renovasc Dis.

[33] Ramos AR, Guilliam D, Dib SI, Koch S (2014). Race/ethnic differences in obstructive sleep apnea risk in patients with acute ischemic strokes in south Florida. Sleep Breath.

[34] Yeh D, Jones M, Schulman C, Karmacharya J, Velazquez OC (2010). Uninsured South Florida vascular surgery patients are less likely to receive optimal medical management than their insured counterparts. J Vasc Surg.

[35] Comuzzie AG, Cole SA, Laston SL, Voruganti VS, Haack K, Gibbs RA, Butte NF (2012). Novel genetic loci identified for the pathophysiology of childhood obesity in the Hispanic population. PLoS One.

[36] Link CL, McKinlay JB (2009). Disparities in the prevalence of diabetes: is it race/ethnicity or socioeconomic status? Results from the Boston Area Community Health (BACH) survey. Ethn Dis.

[37] Fiorentino A, Caivano R, Chiumento C, Cozzolino M, Clemente S, Pedicini P, Fusco V (2012). Comorbidity assessment and adjuvant radiochemotherapy in elderly affected by glioblastoma. Med Oncol.

[38] Mukherjee D, Patil CG, Todnem N, Ugiliweneza B, Nuño M, Kinsman M, Lad SP, Boakye M (2013). Racial disparities in Medicaid patients after brain tumor surgery. J Clin Neurosci.

[39] Curry WT Jr, Carter BS, Barker FG 2nd (2010). Racial, ethnic, and socioeconomic disparities in patient outcomes after craniotomy for tumor in adult patients in the United States, 1988-2004. Neurosurgery.

